# Automated diagnosis of diabetic retinopathy and glaucoma using fundus and OCT images

**DOI:** 10.1186/1476-511X-11-73

**Published:** 2012-06-13

**Authors:** Arulmozhivarman Pachiyappan, Undurti N Das, Tatavarti VSP Murthy, Rao Tatavarti

**Affiliations:** 1 School of Electronics Engineering, VIT University, Vellore, 632014, Tamil Nadu, India; 2 Jawaharlal Nehru Technological University, Kakinada, 533 003, India; 3 UND Life Sciences, 13800 Fairhill Road, #321, Shaker Heights, OH, 44120, USA; 4 Military Hospital, Pune, 411 040, India; 5 GVP-SIRC, GVPCE Campus, Madhurawada, Visakhapatnam, 530048, India

**Keywords:** Fundus image, OCT, Diabetic retinopathy, Glaucoma, RNFL, Image processing

## Abstract

We describe a system for the automated diagnosis of diabetic retinopathy and glaucoma using fundus and optical coherence tomography (OCT) images. Automatic screening will help the doctors to quickly identify the condition of the patient in a more accurate way. The macular abnormalities caused due to diabetic retinopathy can be detected by applying morphological operations, filters and thresholds on the fundus images of the patient. Early detection of glaucoma is done by estimating the Retinal Nerve Fiber Layer (RNFL) thickness from the OCT images of the patient. The RNFL thickness estimation involves the use of active contours based deformable snake algorithm for segmentation of the anterior and posterior boundaries of the retinal nerve fiber layer. The algorithm was tested on a set of 89 fundus images of which 85 were found to have at least mild retinopathy and OCT images of 31 patients out of which 13 were found to be glaucomatous. The accuracy for optical disk detection is found to be 97.75%. The proposed system therefore is accurate, reliable and robust and can be realized.

## Introduction

Diabetic retinopathy (DR) and glaucoma are two most common retinal disorders that are major causes of blindness. DR is a consequence of long-standing hyperglycemia, wherein retinal lesions (exudates and micro aneurysm and hemorrhages) develop that could lead to blindness. It is estimated that 210 million people have diabetes mellitus worldwide [1-3] of which about 10-18% would have had or develop DR [3-6]. Hence, in order to prevent DR and eventual vision loss accurate and early diagnosis of DR is important.

Glaucoma is often, but not always, associated with increased pressure of the vitreous humor in the eye. Glaucoma is becoming an increasingly important cause of blindness, as the world’s population ages [7,8]. It is believed that glaucoma is the second leading cause of blindness globally, after cataract. Both DR and glaucoma are known to be more common in those with hyperlipidemia and glaucoma.

Serious efforts are being made to develop an automatic screening system which can promptly detect DR and glaucoma since early detection and diagnosis aids in prompt treatment and a reduction in the percentage of visual impairment due to these conditions [9-15]. Such an automated diagnostic tool(s) will be particularly useful in health camps especially in rural areas in developing countries where a large population suffering from these diseases goes undiagnosed. We present such an automated system which accepts fundus images and optical coherence tomography (OCT) images as inputs and provides an automated facility for the diagnosis of these diseases and also classify their severity.

**Figure 1:**
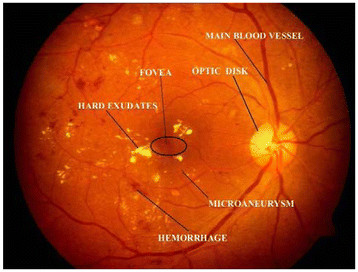
Typical fundus retinal image.

Color fundus images are used by ophthalmologists to study DR. Figure 1 shows a typical retinal image labeled with various feature components of DR. Micro aneurysms appear as small red dots, and may lead to hemorrhage(s); while the hard exudates appear as bright yellow lesions. The spatial distribution of exudates and microaneurysm and hemorrhages, especially in relation to the fovea is generally used to determine the severity of DR.

Ravishankar et al. [16] and others [17-22] showed that blood vessels, exudates, micro aneurysms and hemorrhages can be accurately detected in the images using different image processing algorithms, involving morphological operations. These algorithms first detect the major blood vessels and then use the intersection of these to find the approximate location of the optic disk. Detection of the optic disk, fovea and the blood vessels is used for extracting color information for better lesion detection. But the optical disk segmentation algorithm is rather complex, time consuming, and affected the overall efficiency of the system [23]. In contrast, we describe a simple method that uses fundamental image processing techniques like smoothening and filtering. For this purpose we used the previously described method of dividing the fundus images into ten regions forming fundus coordinates [24] and the presence of lesions in different coordinates was used to determine the severity of the disease [24-28].

Optical coherence tomography (OCT) is an established medical imaging technique. It is widely used, for example, to obtain high-resolution images of the retina and the anterior segment of the eye, which can provide a straightforward method of assessing axonal integrity. This method is also being used by cardiologists seeking to develop methods that uses frequency domain OCT to image coronary arteries in order to detect vulnerable lipid-rich plaques [29,30].

**Figure 2:**
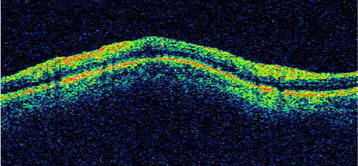
Retinal Nerve Fibre Layer in a typical OCT Image.

Previously, glaucoma was thought to be due to increased intraocular pressure. But, it is now known that glaucoma is also found in people with normal pressure. Glaucoma may lead to damage to optic nerve. The retinal nerve fiber layer (RNFL) when damaged leads to a reduction in its thickness. The diagnosis of glaucoma is arrived at by estimating the thickness of the RNFL. The top red-green region, as shown in Figure 2, is the RNFL region in an OCT image (Figure 2).

The use of Optical Coherence Tomography for diagnosis of glaucoma is a powerful tool. The earlier system with time domain OCT techniques has transformed to a superior system with spectral domain OCT techniques, and has become a well established technique for imaging the depth profile of various organs in medical images [31,32]. Liao *et al.*[33] have used a 2D probability density fields to model their OCT and a level set model to outline the RNFL. They introduced a Kullback-Leiber distance to describe the difference between two density functions that defined an active contours approach to identify the inner and outer boundaries and then a level set approach to identify the retinal nerve fiber layer. Although this technique is successful in determining the thickness, there is an additional requirement of extracting the inner and outer boundaries of the retina prior to identification of the nerve fiber layer. Also they have used separate circular scans to determine the thickness of the RNFL region. On the other hand, Mishra *et al.*[34] have used a two step kernel based optimization scheme to identify the approximate locations of the individual layer, which are then refined to obtain accurate results. However, they have tested their algorithms only on retinal images of rodents.

Speckle noise is inherently present in OCT images and most medical images like ultrasound and MRI. Due to the multiplicative nature of the noise, traditional Gaussian filtering and wiener filtering does not help although they are very robust against additive noise. The use of median filter for de-noising images corrupted with speckle noise is a well established technique in image processing. However, for images corrupted with high degree of speckle, median filtering fails to completely remove the noise. Chan *et al.*[35] have used an iterative gradient descent algorithm, based on progressive minimization of energy to de-noise the speckle corrupted image, and their technique is used in B mode ultrasound imaging. Wong *et al.*[36] suggested a method based on the evaluation of the general Bayesian least square estimate of noise free image, using a conditional posterior sampling approach which was found to be effective for rodent retinal images. Perona and Malik [37] suggested an anisotropic noise suppression technique, in order to deal with this type of noise and also provide edge preservation which is of vital importance in medical image processing where the edges and contours of tissues and organs need to be detected. The smoothing is done locally rather than globally in order to accurately differentiate between the homogenous regions of the ganglions and the boundaries of the RNFL.

Mujat *et al.*[38] have used an active contours based approach to detect the retinal boundaries. Their algorithm uses the multi-resolution deformable snake algorithm and is based on the work of Kass *et al.*[39]. The snake algorithm ensures a search technique which automatically evolves and settles on the contour to be detected. In the present study reported here, we used the anisotropic noise suppression method for dealing with the speckle noise and the greedy snake algorithm [40-43] which provides greater ease of implementation in the discrete domain.

## Materials and methods

A. DR Detection

**Figure 3:**
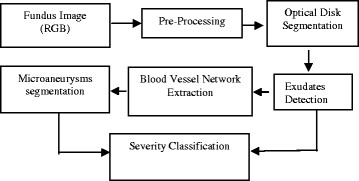
Flow chart for the automated diagnosis of Diabetic Retinopathy using fundus image.

DR detection methodology followed for the extraction of features and classification of severity is given in Figure 3.

1) ***Pre-processing*:** this step involves the illumination equalization and background normalization using adaptive histogram equalization.

2) ***Optical Disk Segmentation and Removal:*** Optical disk detection algorithm uses the property of fundus image that the optical disk region is the brightest region of the fundus image, and therefore the intensity value is the criterion used to detect optical disk. Accordingly, the input RGB image is converted to HSI color plane and I-plane is taken for further processing. Thus, low pass filtering is done on I-plane to smoothen the edges and a threshold criterion is applied on the image. The value of threshold is chosen just below the maximum intensity of fundus image (*I*_max_–0.02, based on our data set of 89 images). After applying the threshold criterion, one may get more than one region. In order to remove other artifacts, a maximum area criterion is used to choose the final optical disk candidate. The region around the final optical disk candidate is segmented to get the region containing optical disk. To detect the boundary of the optical disk, this region is thresholded and optical disk is detected with proper boundary.

3) ***Blood Vessel Extraction:*** Blood vessel extraction is done using morphological closing as described previously [16]. A closing operation is performed on the green channel image using two different sizes of a structuring element (filter). A subtraction of the closed images across two different scales (say, S1 and S2 be the sizes of the structuring elements B1 and B2) will thus give the blood vessel segments C of the green channel image. The image is thresholded and artifacts are removed by eliminating small areas to get the final blood vessel structure.

4) ***Exudates Detection:*** Morphological dilation operation is used to detect exudates [16]. Dilation in gray scale enlarges brighter regions and closes small dark regions. Dilation is performed on the green channel at 2 different scales: S3 and S4, both of which are greater than S2 which was used for vessel extraction. Hence, at both S3 and S4, the blood vessels do not appear in the dilated result.

The exudates being bright with sharp edges respond to dilation. Subtraction of the results across the 2 scales gives the boundaries of the exudates P. The image P is subjected to the threshold criterion to get the binary image P_t_. Morphological filling is performed on P_t_ to get possible optical disk region. The intensity in the green channel image is taken to detect exudates. As the optical disk can also be detected as exudates, the optical disk region coordinates are removed to get final exudates.

5) ***Fovea Detection:*** The fovea is a dark region located in the center of the region of the retina. It commonly appears in microaneurysm and hemorrhage detection results, much as the optic disk does in exudate detection results. The fovea is detected using the location of the optic disk and curvature of the main blood vessel. The main blood vessel is obtained as the thickest and largest blood vessel emanating from the optic disk. The entire course of the main blood vessel is obtained (from the image of the thicker vessels) by looking for its continuity from the optic disk. This blood vessel is modeled as a parabola. The vertex of the parabola is taken as the pixel on the main blood vessel that is closest to the center of the optic disk circular mask. The fovea is located approximately between 2 to 3 optical disk diameter (ODD) distances from the vertex, along the main axis of the modeled parabola and is taken as the darkest pixel in this region. The region of the fovea is taken to be within 1 optic disk diameter of the detected fovea location.

6) ***Micro Aneurysms and Hemorrhages (MAHM) Detection:*** Micro aneurysms are the hardest to detect in retinopathy images. Hemorrhages and micro aneurysms are treated as holes *(i.e.* small dark blobs surrounded by brighter regions) and morphological filling is performed on the green channel to identify them. The unfilled green channel image is then subtracted from the filled one and thresholded in intensity to yield an image (R) with micro aneurysm patches. The threshold is chosen based on the mean intensity of the retinal image in the red channel. Blood vessels can also appear as noise in the microaneurysm and hemorrhage detection as they have color and contrast similar to the clots. Therefore blood vessel coordinates are removed to get final MAHM (micro aneurysms and hemorrhages) candidates.

7) ***Severity Level Classification:*** The distribution of the lesions (exudates and MAHM) about the fovea can be used to predict the severity of diabetic macular edema. As suggested previously [17-23], we divided the fundus image into ten sub-regions about the fovea. The lesions occurring in the macular region are more dangerous and require immediate medical attention, than the ones farther away. As proposed previously [27,28], DR is divided into 5 categories: none, mild, moderate, severe, and proliferative. Our system uses these criteria in order to classify each image in these categories. For performing automated diagnosis of diabetic analysis studies using fundus images a written informed consent was obtained from the patient for publication of this report and other accompanying images.

B. Glaucoma Diagnosis

The estimation of the thickness of the Retinal Nerve Fiber Layer (RNFL) can be broadly broken down into the estimation of the anterior boundary (top layer of RNFL), the posterior boundaries (bottom layer of RNFL) and finally the distance between the two boundaries. The algorithm employed for this purpose is as described previously [38-43]. Two main goals that must be achieved before the thickness of the retinal nerve fibre layer is estimated is the identification of its anterior and the posterior boundaries.

Noise removal is imperative prior to boundary detection. Any imaging technique which is based on detection of coherent waves is affected by speckle noise. Since OCT is also based on interferometric detection of coherent optical beams, OCT images contain speckle noise. The speckle noise is multiplicative in nature which implies that it is an implicit composition of the information and the noise. The major challenge that needs to be tackled while reducing the effect of speckle noise is minimizing the loss of relevant details like the edges. Noise reduction algorithms with edge preservation thus become an optimal choice in such situations. These not only improve the visual appearance of the image, but also potentially improve the performance of subsequent boundary detection algorithm. In the present study, we employed the anisotropic noise suppression technique [37,38], which smoothes the image but at the same time preserve the edges. The next major step is the estimation of the anterior and the posterior boundaries. This is done using the deformable snake algorithm [39-43]. This is an iterative process which identifies the points with the maximum gradient, thereby detecting the boundary. 

1) Anterior Boundary Estimation

Prior to estimation of the anterior boundary, the image is first smoothed using a 10 × 10 Gaussian kernel and standard deviation of 4. The image is then filtered using a 3 × 3 median filter which is very effective against speckle noise. The next step is to find an initial estimate of the anterior layer, which evolves as per the snake algorithm [39-43]. The initial estimate is found by first binarizing the magnitude of the image gradient. The estimate is then found as the first white pixel from the top. However, sometimes there are holes in the anterior boundary and the first pixel identified may not be on the anterior layer. This means that there are still some white pixels that need to be removed. This is done by removing the white pixels which have area less than 158 pixels (0.07% of the total image size [38]. Also any connected region, which is less than 25 pixels in length, is removed. These two morphological operations ensure that the white pixels are only those of the anterior boundary. Next we fill in the holes in the anterior boundary using a cubic polynomial curve fitting scheme. In this, using the set of points which lie on the anterior boundary, a cubic polynomial is generated. Using this polynomial equation the missing pixels can then be identified for every column.

2) ***Posterior Boundary Estimation*:** The posterior boundary estimation requires a few more pre-processing steps. First, everything above the anterior boundary is removed. Next a noise removal technique is employed prior to extraction of the posterior boundary so that a relatively more accurate estimate can be obtained. The joint anisotropic noise suppression algorithm with edge preservation is implemented as suggested by Perona and Malik [37].

The equation for anisotropic noise suppression involves the calculation of the divergence of the sum of the Laplacian and the gradient of the image. The output of this image is an image which is smoothed, except at the boundaries. In discrete domain, it also includes a time factor which is incorporated from its analogy to the heat diffusion process. The equation is implemented in discrete domain as follows:

(1) $$I_{\tau +1}=I_{\tau }+\lambda \left(c_{N}\cdot \nabla_{N}I+c_{S}\cdot \nabla_{S}I+c_{E}\cdot \nabla_{E}I+c_{W}\cdot \nabla_{W}I+c_{\mathrm{NE}}\cdot \nabla_{\mathrm{NE}}I+c_{\mathrm{NW}}\cdot \nabla_{\mathrm{NW}}I+c_{\mathrm{SE}}\cdot \nabla_{\mathrm{SE}}I+c_{\mathrm{SE}}\cdot \nabla_{\mathrm{SE}}I\right)$$

The subscripts *N, S, E, W, NE, NW, SE,* and *SW* correspond to the neighborhood pixels. Although the original work of Perona and Malik [37] describes the use of only 4 neighbors, the use of eight neighbors in our algorithm has been found to be particularly more effective. The value of λ can be chosen as any value between 0 and 0.25. Here the symbol ∇ represents the Laplacian and is calculated in discrete domain as follows:

(2) $$\nabla_{N}I_{i,j}=I_{i-1,j}-I_{i,j}$$

(3) $$\nabla_{S}I_{i,j}=I_{i+1,j}-I_{i,j}$$

(4) $$\nabla_{E}I_{i,j}=I_{i,j+1}-I_{i,j}$$

(5) $$\nabla_{W}I_{i,j}=I_{i,j-1}-I_{i,j}$$

(6) $$\nabla_{\mathrm{NE}}I_{i,j}=I_{i-1,j+1}-I_{i,j}$$

(7) $$\nabla_{\mathrm{NW}}I_{i,j}=I_{i-1,j-1}-I_{i,j}$$

(8) $$\nabla_{\mathrm{SE}}I_{i,j}=I_{i+1,j+1}-I_{i,j}$$

(9) $$\nabla_{\mathrm{SW}}I_{i,j}=I_{i+1,j-1}-I_{i,j}$$

The value of the conduction coefficient *C* is updated after every iteration, as a function of the image intensity gradient.

(10) $$C_{N}=g\left(\left\|I_{i-1,j}\right\|\right)$$

(11) $$C_{S}=g\left(\left\|I_{i+1,j}\right\|\right)$$

(12) $$C_{E}=g\left(\left\|I_{i,j+1}\right\|\right)$$

(13) $$C_{W}=g\left(\left\|I_{i,j-1}\right\|\right)$$

(14) $$C_{\mathrm{NE}}=g\left(\left\|I_{i-1,j+1}\right\|\right)$$

(15) $$C_{\mathrm{NW}}=g\left(\left\|I_{i-1,j-1}\right\|\right)$$

(16) $$C_{\mathrm{SE}}=g\left(\left\|I_{i+1,j+1}\right\|\right)$$

(17) $$C_{\mathrm{SW}}=g\left(\left\|I_{i+1,j-1}\right\|\right)$$

There are two choices of the function *g*[37].The first of the two equations described by Perona and Malik [37], preserves high contrast edges over low contrast edges, while the second one preserves wide regions over smaller ones. Since our aim is to detect the boundary we choose the first function which is mentioned below again for convenience.

(18) $$g\left(\nabla I\right)=e^{\left(-{\left(\left\|\left(\nabla I|K\right)\right\|\right)}^{2}\right)}$$

The constant *K* is chosen statistically to give perceptually best results. Once the noise suppression algorithm has been implemented the extraction of the posterior boundary becomes fairly simple since the portions of the interior of the RNFL get smoothed and the posterior boundary becomes much more distinct. An edge field is calculated by first finding the magnitude of the image gradient of the smoothed field obtained as a result of the joint anisotropic noise suppression algorithm. Then the image is first normalized and then binarized using a suitable threshold which is set statistically. Once this has been done there are still some areas which contain some unwanted white portions which are removed by removing those portions which have a pixel area of less than 100. Next the regions from below, the nerve fiber layer are eliminated which basically consist of the Retinal Pigment Epithelium (RPE). Also the anterior boundary is removed completely. However, there are still certain disconnected regions which were a part of RPE or the anterior boundary remain and need to be removed. This is done by removing areas having length less than 25 pixels and also areas which are less than 70 pixels [38]. The posterior boundary is then estimated as the first white pixel from the top. The points extracted are then passed through a median filter of 50 points in order to remove any unwanted spikes. This completes the detection of the posterior boundary. Now both the anterior and the posterior boundaries have been identified and the thickness is determined as the pixel difference between the boundaries. The thickness of each pixel depends on the OCT acquisition mechanism. In our case the pixel thickness is 6 μm. The thickness at each point of the anterior and posterior boundaries is calculated and then averaged over the length of the image. For performing automated diagnosis of Glaucoma studies using OCT images a written informed consent was obtained from the patient for publication of this report and other accompanying images.

## Results and discussion

A. DR Diagnosis

**Figure 4:**
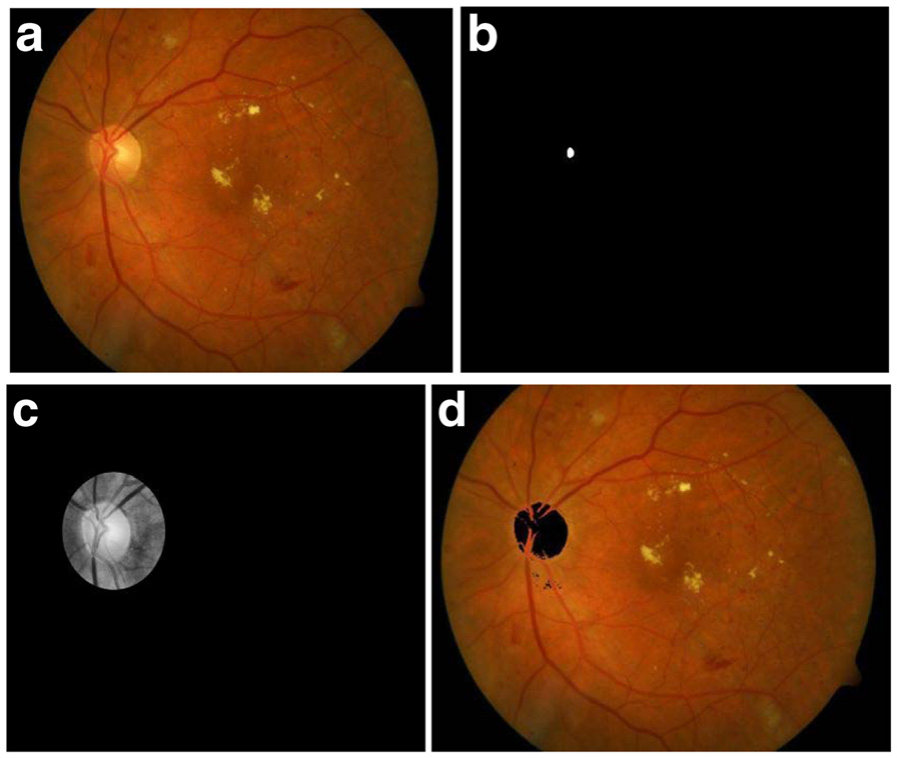
**Optical Disc detection process.** (**a**) Input fundus image, (**b**) Optical Disc localization, (**c**) Optical Disc region, (**d**) Optical Disc detected.

**Figure 5:**
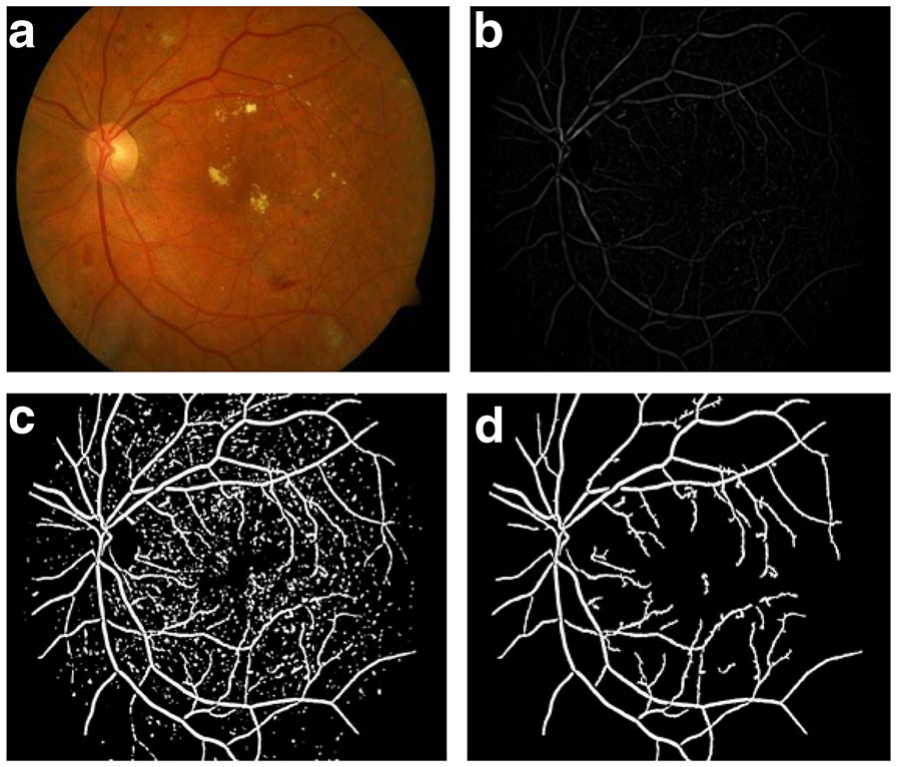
**Blood vessel detection process.** (**a**) Input fundus image, (**b**) Fundus gradient image, (**c**) Thresholded fundus gradient image, (**d**) Blood vessels detected.

**Figure 6:**
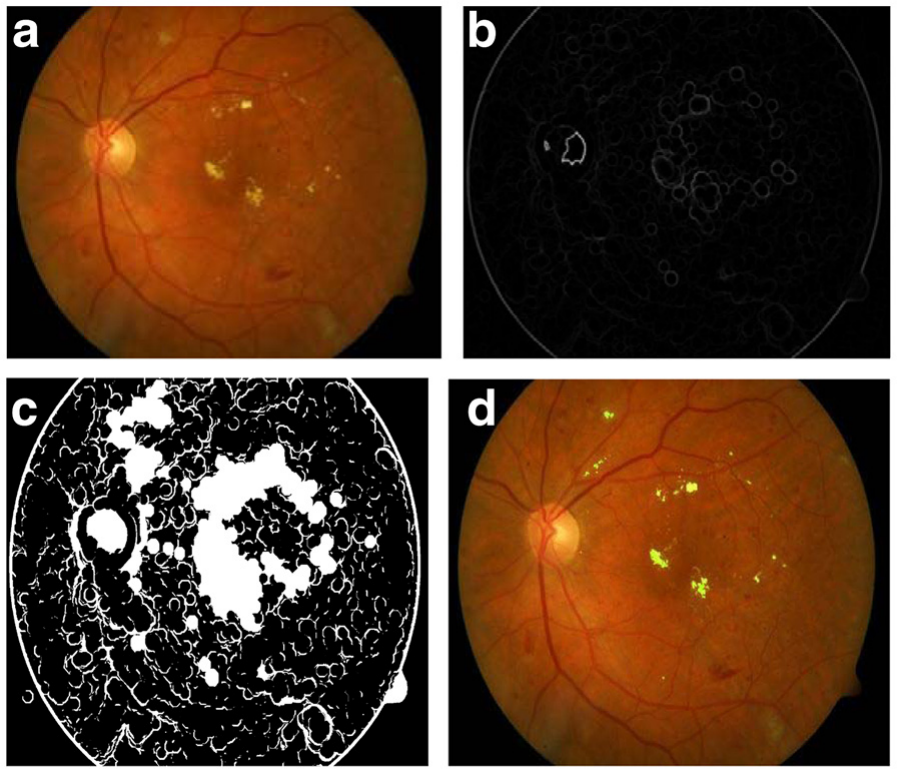
(a) Input Fundus Image, (b) Dilation gradient image, (c) Thresholded and filled image, (d) Exudates detected.

**Figure 7:**
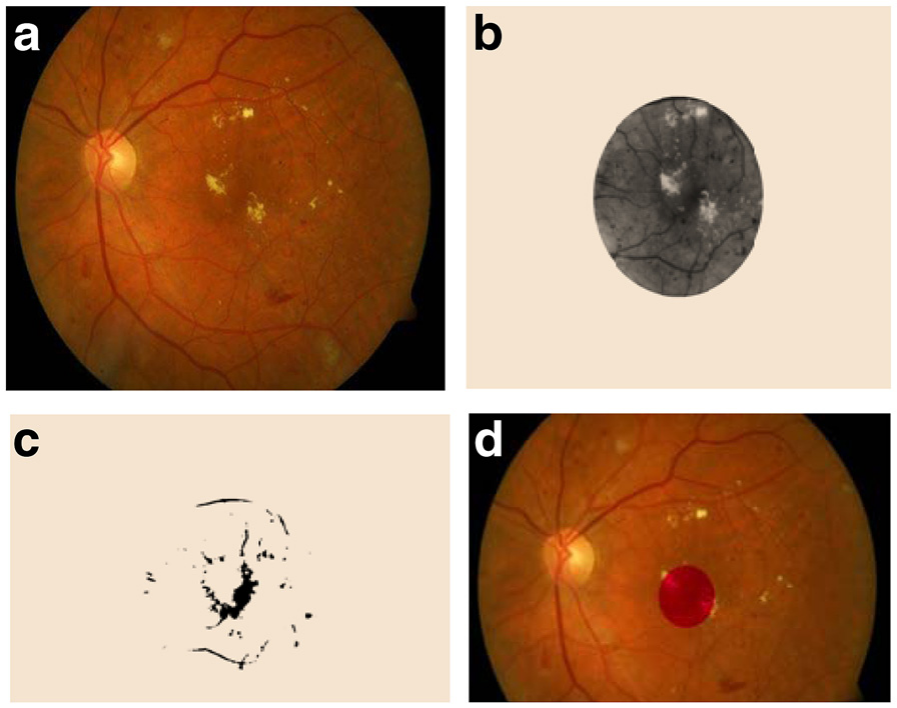
(a) Input Fundus Image, (b) Possible Fovea region, (c) Threshold region, (d) Fovea detected.

**Figure 8:**
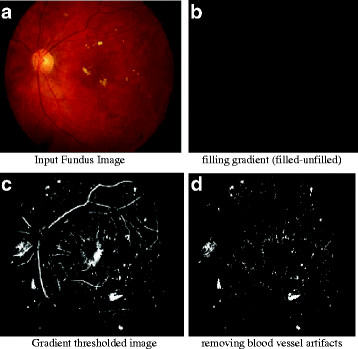
(a) Input Fundus Image, (b) filling gradient (filled-unfilled), (c) Gradient thresholded image, (d) removing blood vessel artifacts.

**Figure 9:**
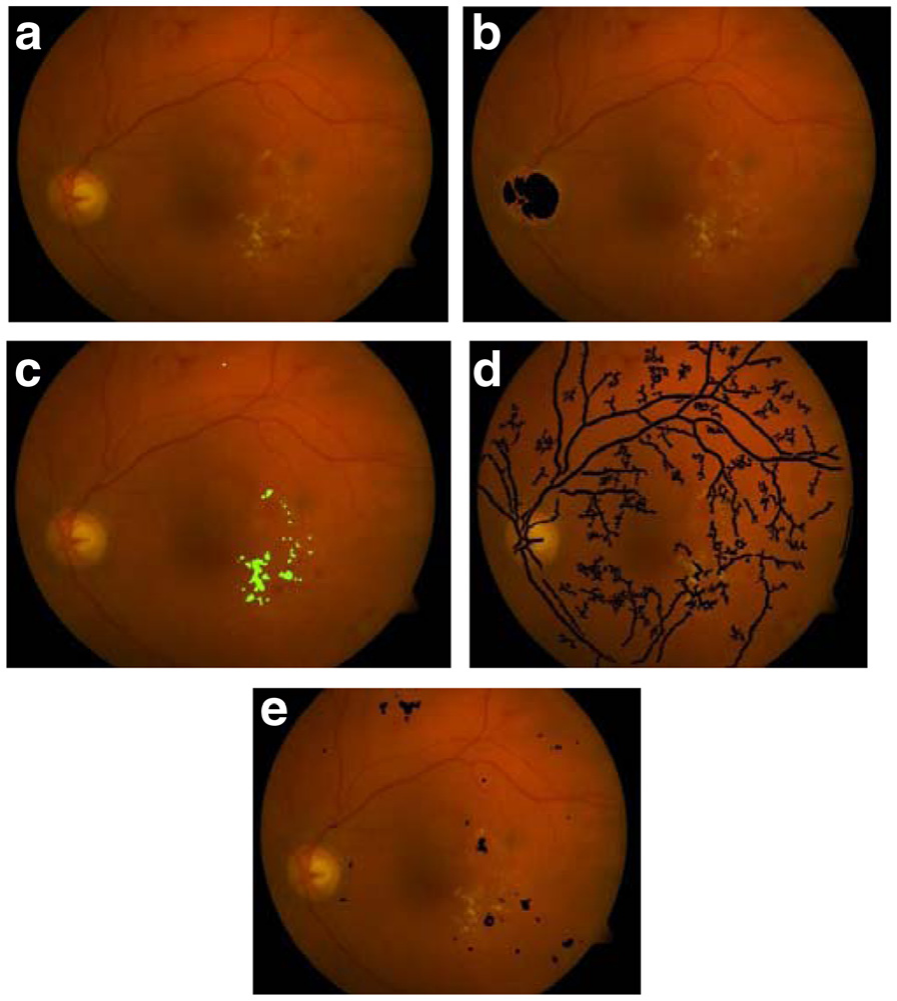
**Total exudate area for above patient is 5196 pixels, total MAHM area is 3991 pixels and there is no exudate and MAHM pixel in fovea.** Therefore the DR condition is classified as moderate. (**a**) Input RGB fundus image, (**b**) Optical Disk Detected, (**c**) Exudates Detection, (**d**) Blood vessel segmentation, (**e**) MAHM detected.

The results were obtained for eight nine (89) fundus images [44] which were used for detection and diagnosis of DR. The individual segmentation modules were developed using MATLAB, later integrated to act as standalone application software. The segmentation of Micro Aneurysms, Hard Exudates, Cotton Wool Spots, Optic Disc, and Fovea was successfully performed and the results obtained show high degree of accuracy, independent of different coordinates of the retinal Angiogram datasets. Some of the results obtained for the diagnosis of DR are shown in Figures 3, 4, 5, 6, 7 and 8. The total area occupied and the area occupied in the fovea region is calculated corresponding to the exudates and micro aneurysms, based on the number of pixels and the severity level was determined as none, mild, moderate and severe. Figure 9 shows the results of DR diagnosis of a typical patient, based on the fundus image. 

B. Glaucoma Diagnosis

**Figure 10:**
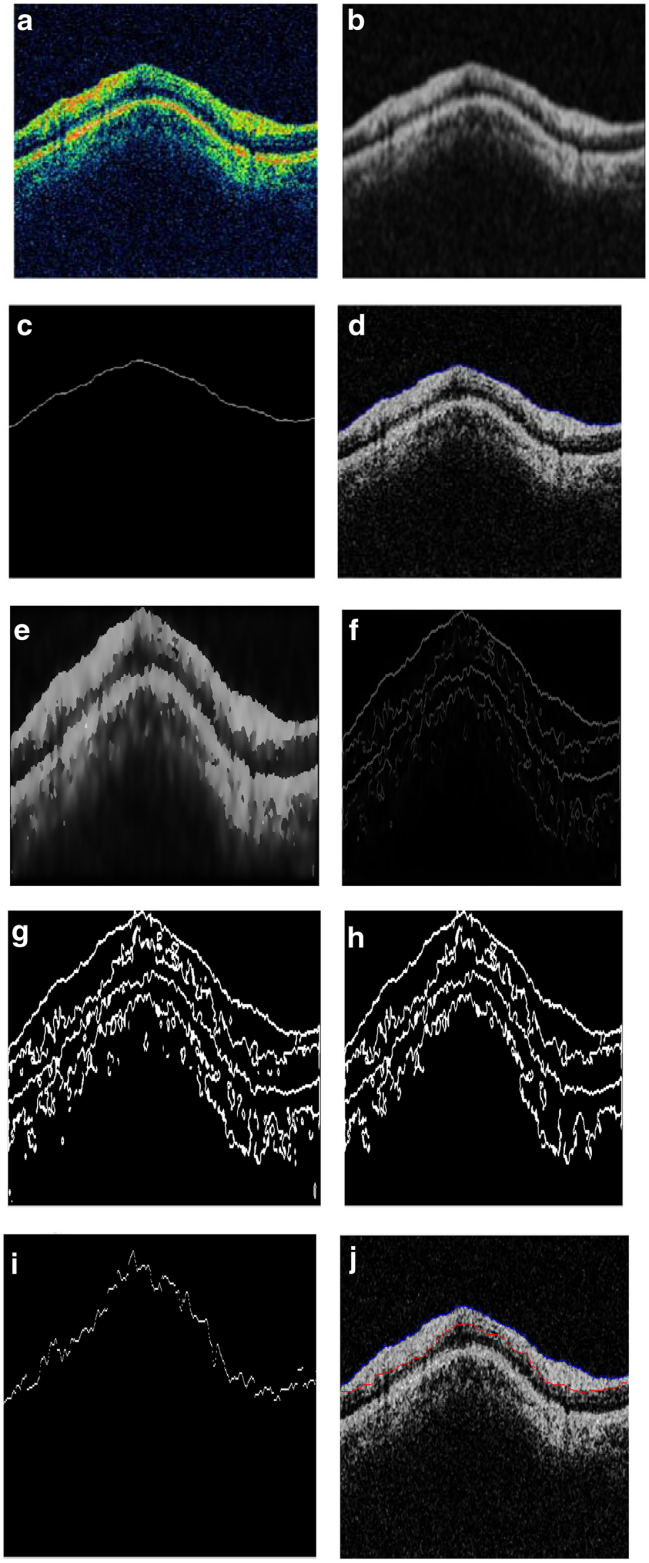
(a) input OCT image; (b) Gaussian smoothed median filtered image; (c) initial estimate of the anterior boundary; (d) accurately detected anterior boundary after applying snake algorithm; (e) Smoothed image with edges preserved using anisotropic diffusion; (f) edge field of image in 10(e); (g) binarized version of image in 10(f); (h) areas less than 100 pixels are removed; (i) initial estimate of Posterior Boundary; (j) Accurately detected posterior boundary.

**Figure 11:**
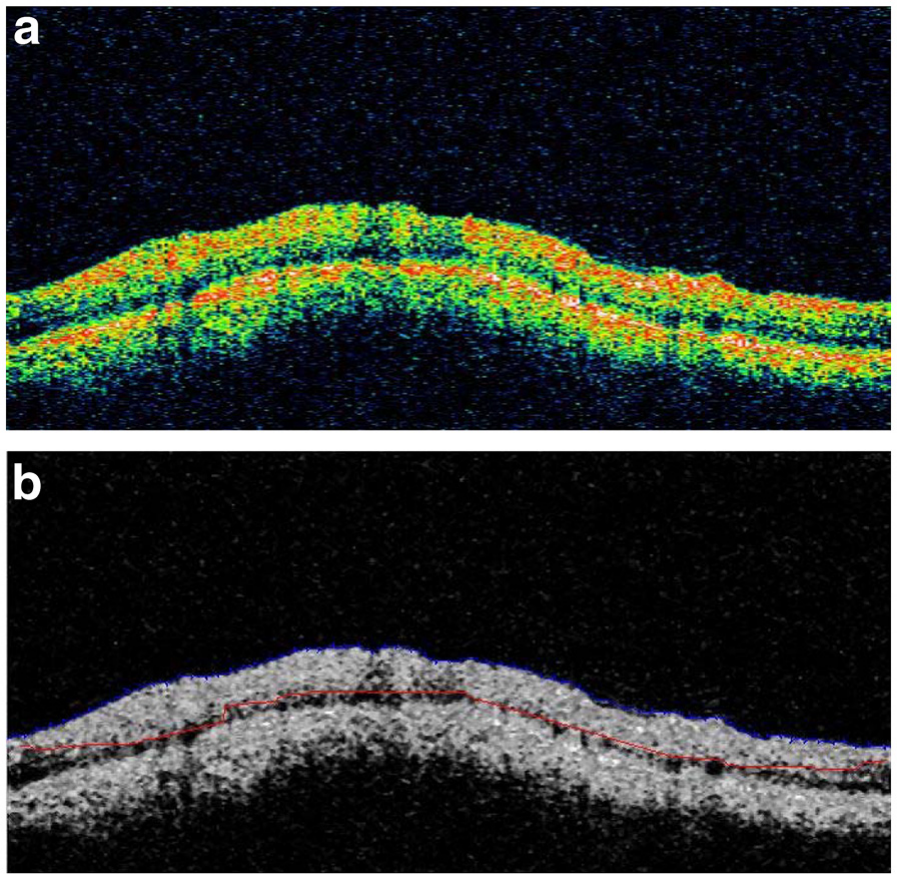
(a): Input OCT image, (b): Anterior and posterior boundaries in blue and red respectively.

Figure 10 (a-f) shows the steps described above with respect to Glaucoma diagnosis - starting from the initial estimate of the anterior boundary to detection of both the boundaries. The algorithm for the diagnosis of Glaucoma by measurement of the retinal nerve fiber layer thickness was tested on a set of 186 images of 31 patients *i.e*.*,* three images each of the right and the left eye. The mean thickness for both the eyes was calculated and the classification into Glaucomatous and Non-Glaucomatous was done based on whether the thickness of the nerve fiber layer is lesser or greater than 105 μm [45,46]. The images are of the dimension 329 × 689pixels. The algorithm was implemented using Matlab 7.10 on an *Intel Core2 Duo Processor 2.2 GHz* machine. The results are shown in Figure 11. Figure 11 shows the input OCT image and the corresponding output image of a typical patient. Out of the 31 patients, 13 patients were found to have glaucoma in at least one eye; *i.e.,* their RNFL thickness was less than 105 μm. The image shown above has an RNFL thickness of 168.06 μm, indicating a healthy candidate. 

## Conclusions

Here we have described a low cost retinal diagnosis system which can aid an ophthalmologist to quickly diagnose various stages of diabetic retinopathies and glaucoma. This novel system can accept both kinds of retinal images (fundus and OCT) and can successfully detect any pathological condition associated with retina. Such a system can be of significant benefit for mass diagnosis in rural areas especially in India where patient to ophthalmologist ratio is as high as (4,00,000:1) [47]. A major advantage of our algorithm is that the accuracy achieved for optical disk detection is as high as 97.75% which implies greater accuracy of exudates detection. Our results show that RNFL thickness measurement using our proposed method is concurrent with the ophthalmologist’s opinion for glaucoma diagnosis. This work can be extended to develop similar diagnostic tools for other ocular diseases and combining it with telemedicine application, for remote, inaccessible and rural areas may prove to be of significant benefit to diagnose various retinal diseases.

Furthermore, it is also relevant to note that the risk of development of both diabetic retinopathy and glaucoma are enhanced in those with hyperlipidemia [48,49]. This suggests that whenever diabetic retinopathy and glaucoma are detected in a subject they also should be screened for the existence of hyperlipidemia. Thus, early detection of diabetic retinopathy and glaucoma may also form a basis for screening of possible presence of dyslipidemia in these subjects. In this context, it is important to note that type 2 diabetes mellitus, glaucoma and hyperlipidemia are all considered as low-grade systemic inflammatory conditions [50,51] providing yet another reason as to why patients with DR and glaucoma need to be screened for hyperlipidemia.

## Competing interest

The authors declare that they have no competing interests.

## Authors’ contributions

PA carried out experimental studies on automated diagnosis of diabetic retinopathy and glaucoma studies using fundus and OCT images, PA, UND, TVSPM and RT participated in the sequence of algorithm studies and interpretation of results and interaction with ophthalmologists also all the authors participated in the sequence alignment and drafted the manuscript. All authors read and approved the final manuscript.
